# Sex differences in intracranial and extracranial atherosclerosis in patients with acute ischemic stroke

**DOI:** 10.1177/1747493020932806

**Published:** 2020-09-02

**Authors:** S Voigt, HJA van Os, MAA van Walderveen, IC van der Schaaf, LJ Kappelle, A Broersen, BK Velthuis, PA de Jong, R Kockelkoren, ND Kruyt, A Algra, MJH Wermer, on behalf of the DUST study group

**Affiliations:** 1 Department of Neurology, 4501 Leiden University Medical Center, Leiden, the Netherlands; 2 Department of Radiology, 4501 Leiden University Medical Center, Leiden, the Netherlands; 3 Department of Radiology, University Medical Center Utrecht, Utrecht, the Netherlands; 4 Department of Neurology and Neurosurgery, University Medical Center Utrecht and Utrecht University, Utrecht, the Netherlands; 5 Julius Center for Health Sciences and Primary Care, University Medical Center Utrecht, Utrecht, the Netherlands

**Keywords:** Computed tomography scan, ischemic stroke, intracranial atherosclerosis, extracranial atherosclerosis, sex differences, stroke

## Abstract

**Background and aim:**

To investigate sex differences with respect to presence and location of atherosclerosis in acute ischemic stroke patients.

**Methods:**

Participants with acute ischemic stroke were included from the Dutch acute stroke trial, a large prospective multicenter cohort study performed between May 2009 and August 2013. All patients received computed tomography/computed tomography-angiography within 9 h of stroke onset. We assessed presence of atherosclerosis in the intra- and extracranial internal carotid and vertebrobasilar arteries. In addition, we determined the burden of intracranial atherosclerosis by quantifying internal carotid and vertebrobasilar artery calcifications, resulting in calcium volumes. Prevalence ratios between women and men were calculated with Poisson regression analysis and adjusted prevalence ratio for potential confounders (age, hypertension, hyperlipidemia, diabetes, smoking, and alcohol use).

**Results:**

We included 1397 patients with a mean age of 67 years, of whom 600 (43%) were women. Presence of atherosclerosis in intracranial vessel segments was found as frequently in women as in men (71% versus 72%, adjusted prevalence ratio 0.95; 95% CI 0.89–1.01). In addition, intracranial calcification volume did not differ between women and men in both intracranial internal carotid (large burden 35% versus 33%, adjusted prevalence ratio 0.93; 95% CI 0.73–1.19) and vertebrobasilar arteries (large burden 26% versus 40%, adjusted prevalence ratio 0.69; 95% CI 0.41–1.12). Extracranial atherosclerosis was less common in women than in men (74% versus 81%, adjusted prevalence ratio 0.86; 95% CI 0.81–0.92).

**Conclusions:**

In patients with acute ischemic stroke the prevalence of intracranial atherosclerosis does not differ between women and men, while extracranial atherosclerosis is less often present in women compared with men.

## Introduction

Atherosclerosis is the most common cause of ischemic stroke worldwide and an important prognostic factor for recurrent vascular events.^1^ Evidence is accumulating that development, distribution, and severity of atherosclerosis may be dependent on sex.^2,3^ Several studies have shown that extracranial atherosclerosis is more common in men than in women with ischemic stroke.^4^ Less is known about sex differences in intracranial atherosclerosis. Risk factors for intracranial internal carotid artery (ICA) calcification seem to differ between men and women, suggesting a difference in pathophysiological mechanisms.^2^

Studies on sex differences in intracranial atherosclerosis have been mainly performed in Asian populations. One large Chinese stroke study with 1335 participants found no sex differences in presence of intracranial atherosclerosis.^5^ In contrast, in another Chinese stroke study with 551 participants, men had a higher prevalence of intracranial atherosclerosis with an odds ratio of 2.3 (95% confidence interval (CI): 1.48–3.26).^6^ Studies on intracranial atherosclerosis in Caucasian stroke patients are scarce.

We investigated sex differences in presence, location, and burden of intra- and extracranial atherosclerosis in a large population of Western-European acute ischemic stroke patients.

## Methods

### Participants

We included participants from the Dutch Acute Stroke Trial (DUST), a large prospective multicenter cohort study performed between May 2009 and August 2013. The aim of DUST was to investigate the value of CT-angiography (CTA) and CT-perfusion (CTP) for predicting outcome after ischemic stroke. Inclusion criteria were age ≥18 years, onset of stroke symptoms <9 h, and National Institute of Health Stroke Scale (NIHSS) ≥2 or ≥1 if intravenous thrombolysis was indicated. Exclusion criteria were known renal failure and contrast agent allergy. For the current study, participants from all 14 participating DUST centers were included.^7^

We retrieved the following characteristics on admission from the CRFs of the DUST: demographic features, cardiovascular risk factors, a history of cardiovascular disease, baseline NIHSS, and blood pressure on admission. Stroke etiology was classified according to the TOAST criteria. All participants underwent non-contrast computed tomography (NCCT), CTA, and CTP on admission with scan protocols standardized between centers.^7^

### Standard protocol approvals, registrations, and patient consents

DUST was approved by the Medical Ethics Committee of the University Medical Center Utrecht and local approval was obtained from all participating hospitals. CTA/CTP imaging on admission was performed as part of the routine clinical work-up of ischemic stroke patients. Patients or a legal representative gave written informed consent for use of clinical and imaging follow-up data. The medical ethics committee waived the need for informed consent for patients who died before consent could be obtained.

### Radiologic parameters

Scans were assessed by a certified neuro-radiologist with at least five years of experience (from a pool of three observers). We assessed the presence of any sign of atherosclerosis (both non-calcified and calcified plaque on CTA) in intracranial and extracranial segments of the ICA or VBA.

In addition, we determined the burden of calcified intracranial atherosclerosis by assessing intracranial ICA and vertebrobasilar artery (VBA) calcifications on thin slice (0.5–0.9 mm) NCCT, using calcium as measure for intracranial atherosclerosis since both parameters are strongly related.^8^ Intracranial ICA calcifications were measured manually from the petrous bone to the top of the intracranial ICA, and VBA calcifications were assessed from the point where the vertebral arteries enter the dura until the tip of the basilar artery, at the origin of the posterior cerebral arteries. Calcium volumes were assessed using dedicated software (CalcScore V11.1 by Medis Specials bv).^9^ Regions of interest were drawn to discern the intracranial ICA calcifications from the skull base using a threshold, which was set to the optimal threshold of 160 Hounsfield units (HU) by performing a small pilot study.^9^ Since calcification volumes were non-normally distributed we divided ICA and VBA calcification volumes into tertiles. We then analyzed large (upper tertile) versus small (middle and lower two tertiles) burden of intracranial atherosclerosis. We only trichotomized calcification volumes for patients in whom VBA calcifications were present, as the majority of patients did not have any VBA calcifications. We followed the same procedure for ICA calcifications.

Intracranial ICA calcifications were also subdivided according to dominant intimal or dominant medial location. This was done using a previously validated score based on matched NCCT- and histological slides.^10^ The score was constructed by assigned points to different calcification characteristics (circularity, thickness, and morphology) which were weighted according to their relation to either medial or intimal calcification.^3^

Extracranial vessel segments were divided into anterior (common and internal carotid arteries) and posterior (vertebral arteries). On CTA, we defined stenosis of the extracranial circulation as any sign of stenosis and stenosis of ≥70%, performed according to the NASCET criteria.

### Statistical analysis

We performed (multivariable) Poisson regression analyses to identify possible relationships between sex and radiological characteristics of atherosclerosis.^11^ Adjustments were made for age, hypertension, hyperlipidemia, diabetes, smoking, and alcohol use because we considered these factors to be potential confounders. Additionally, we stratified for age (young stroke defined as stroke <50 years). Prevalence ratios (PRs) and adjusted PR (aPR) with 95% CI were calculated. For all analyses we performed complete case analysis. All data were analyzed with SPSS (v25).

## Results

In total, 1397 participants were included. Mean age was 67 ± 14 (SD) years, 600 (43%) were women, and median NIHSS was 6 (Table 1).

**Table 1.** Clinical characteristics of the participants

Characteristics	Women (n = 600, 43%)	Men (n = 797, 57%)
Demographics		
Age, mean years (±SD)	69 (15.4)	66 (13.0)
History, n (%)		
Hypertension	331 (56%)	397 (50%)
Diabetes mellitus	93 (16%)	121 (15%)
Hyperlipidemia	181 (31%)	276 (36%)
Previous stroke or TIA	155 (26%)	187 (24%)
Myocardial infarction	41 (7%)	129 (17%)
Atrial fibrillation	69 (12%)	109 (14%)
Peripheral artery disease	27 (5%)	55 (7%)
Smoking		
Current	134 (25%)	249 (34%)
Lifetime^a^	148 (27%)	265 (36%)
Alcohol use	183 (31%)	347 (44%)
Baseline NIHSS,^b^ median	6	6
Blood pressure on admission		
Systolic BP > 160 mm Hg	226 (38%)	296 (37%)
Diastolic BP > 90 mm Hg	158 (26%)	276 (35%)
Stroke etiology	n = 380	n = 561
Large vessel disease	171 (45%)	249 (45%)
Cardiac embolic source	99 (26%)	136 (24%)
Small vessel disease/lacunar infarct	80 (21%)	103 (18%)
Dissection	10 (3%)	40 (7%)
Other	20 (5%)	33 (6%)

BP: blood pressure; NIHSS: National Institute of Health Stroke Scale; TIA: Transient Ischemic Attack.

a Current smokers and smokers who stopped smoking >6 months ago.

b Baseline NIHSS: NIH Stroke Scale on admission.

### Intracranial atherosclerosis

The overall prevalence of any intracranial atherosclerosis was 72%. Presence of atherosclerosis in intracranial vessel segments was found as frequently in women as in men (71% versus 72%, aPR: 0.95; 95% CI 0.89–1.01) (Table 2).

**Table 2.** Atherosclerotic characteristics in women and men

Atherosclerotic characteristics	Women (n = 600)	Men (n = 797)	PR (95% CI)	aPR (95% CI)^a^
Intracranial circulation (ICA, VBA, MCA, ACA, and PCA)				
Any sign of atherosclerosis (n = 1369)	416 (71%)	565 (72%)	0.98 (0.91–1.04)	0.95 (0.89–1.01)
Large versus small burden ICA calcification volume (n = 873)^b^	126 (35%)	166 (33%)	1.07 (0.84–1.34)	0.93 (0.73–1.19)
Large versus small burden VBA calcification volume (n = 250)b	27 (26%)	58 (40%)	0.66 (0.41–1.04)	0.69 (0.41–1.12)
Any sign of stenosis (n = 1182)	69 (12%)	120 (15%)	0.76 (0.57–1.00)	0.68 (0.50–0.93)
Intima ICA calcification (n = 1132)	123 (25%)	228 (36%)	0.71 (0.59–0.85)	0.76 (0.63–0.93)
Media ICA calcification (n = 1132)	262 (54%)	269 (42%)	1.28 (1.13–1.45)	1.09 (0.96–1.25)
Extracranial circulation				
Any sign of atherosclerosis (n = 1371)	433 (74%)	631 (81%)	0.91 (0.86–0.97)	0.86 (0.81–0.92)
Atherosclerosis anterior circulation (n = 1371)	426 (72%)	617 (79%)	0.92 (0.86–0.98)	0.87 (0.82–0.93)
Atherosclerosis posterior circulation (n = 1360)	202 (34%)	309 (40%)	0.85 (0.74–0.98)	0.77 (0.66–0.89)
Any sign of stenosis (n = 1396)	244 (41%)	378 (47%)	0.86 (0.76–0.97)	0.78 (0.68–0.89)
Stenosis ≥ 70% (n = 1396)	100 (17%)	213 (27%)	0.63 (0.51–0.77)	0.60 (0.47–0.76)

ACA: Anterior Cerebral Artery; aPR: adjusted prevalence ratio; CI: confidence interval; ICA: intracranial carotid artery; MCA: Middle Cerebral Artery; PCA: Posterior Cerebral Artery; PR: prevalence ratio; VBA: vertebrobasilar artery.

a Adjusted for age, hypertension, hyperlipidemia, diabetes, smoking, and alcohol use.

b Tertiles are based on the patients with presence of ICA (n = 873) or VBA (n = 250) calcifications, respectively. We analyzed large (upper tertile) versus small (middle and lower two tertiles) burden of intracranial atherosclerosis.

In 914 of the 1397 patients, the thin-slice NCCT was retrievable and of sufficient quality for calcification volume measurements. The baseline characteristics of the included and excluded patients were similar (Supplementary Table 1). In 11 of these scans only assessment of ICA calcification volume was possible, as an artifact in the posterior fossa prohibited assessment of VBA calcification volume. Intracranial ICA calcification volume did not differ between women and men in the ICA (large burden 35% versus 33%, aPR 0.93; 95% CI 0.73–1.19) or the VBAs (large burden 26% versus 40%, aPR 0.69; 95% CI 0.41–1.12). Intima calcifications in the intracranial circulation were less prevalent in women (25% versus 36%, aPR 0.76; 95% CI 0.63–0.93), whereas for presence of media calcifications no clear sex differences were found after adjustments (54% versus 42%, aPR 1.09; 95% CI 0.96–1.25).

In the 180 participants younger than 50 years (50% women), the presence and volume of intracranial atherosclerosis was similar in men and women (Table 3).

**Table 3.** Any sign of atherosclerotic changes according to sex difference, stratified for age

Subgroups	Atherosclerotic characteristics	Women (n = 598)	Men (n = 795)	PR (95% CI)	aPR (95% CI)^a^
Age group < 50 (n = 180)		n = 90	n = 90		
Intracranial circulation	Any sign of intracranial atherosclerosis	17 (20%)	14 (16%)	1.23 (0.66–2.39)	0.97 (0.52–1.83)
	Intima calcification	9 (12%)	11 (16%)	0.76 (0.34–1.73)	0.67 (0.30–1.49)
	Media calcification	9 (12%)	8 (12%)	1.05 (0.43–2.56)	1.04 (0.44–2.49)
Extracranial circulation	Any sign of extracranial atherosclerosis	23 (27%)	21 (24%)	1.13 (0.68–1.89)	0.94 (0.55–1.60)
	Stenosis ≥ 70%	13 (15%)	17 (19%)	0.77 (0.40–1.50)	0.78 (0.40–1.51)
Age group ≥ 50 (n = 1213)		n = 508	n = 705		
Intracranial circulation	Any sign of intracranial atherosclerosis	398 (79%)	550 (80%)	0.99 (0.94–1.05)	0.99 (0.93–1.05)
	Intima calcification	114 (28%)	217 (38%)	0.73 (0.60–0.88)	0.85 (0.69–1.03)
	Media calcification	253 (61%)	261 (46%)	1.34 (1.19–1.51)	1.12 (0.98–1.28)
Extracranial circulation	Any sign of extracranial atherosclerosis	410 (82%)	609 (88%)	0.93 (0.89–0.98)	0.90 (0.85–0.95)
	Stenosis ≥70%	31 (6%)	54 (8%)	0.96 (0.90–1.02)	0.96 (0.90–1.02)

aPR: adjusted prevalence ratio; CI: confidence interval.

a Adjusted for age, hypertension, hyperlipidemia, diabetes, smoking, and alcohol use.

### Extracranial atherosclerosis

In extracranial vessels, women with stroke less often had any sign of atherosclerosis (74% versus 81%, aPR 0.86; 95% CI 0.81–0.92) and less often a significant stenosis ≥70% than men (17% versus 27%, aPR 0.60; 95% CI 0.47–0.76) (Table 2).

## Discussion

In our study the prevalence and extent of atherosclerotic changes of the intracranial circulation in patients admitted for stroke was comparable in men and women whereas women less often had signs of extracranial atherosclerosis than men.

The prevalence of intracranial ICA calcifications in our population was 72%. A study of 406 Dutch patients (mean age of 62 years) suspected of TIA or minor stroke reported a prevalence of intracranial ICA calcifications of 65%.^12^ In this study a significantly higher threshold (500 HU) was used for detection of calcifications.^12^ The prevalence of intracranial ICA calcifications in the Rotterdam population based study (N = 2495, mean age 70) was 82%.^13^ Possibly, the lower threshold for differentiation of calcifications (130 HU) as well as the higher mean age could explain the difference between the Rotterdam study and DUST.^13^ Our threshold of 160 HU was based on optimal distinction between intracranial ICA calcifications and skull base in data from all different vendors; calcification volume data did not differ notably between centers. In a previous Chinese study the prevalence of intracranial atherosclerosis was 63% in a population of ischemic stroke patients.^6^ This lower prevalence may be caused by ethnical differences.^14^

It has been previously reported that extracranial circulation atherosclerosis is more prevalent in men than in women.^4^ Common understanding is that this is due to lack of protective effect of estrogen or increased exposure to vascular risk factors.^15^ In contrast to the sex differences in the extracranial circulation in our study, intracranial atherosclerosis prevalence was similar in men and women. Risk factors for intracranial ICA calcification seem to differ between men and women, suggesting a difference in pathophysiological mechanisms.^2^ Excessive alcohol intake and smoking have been associated with intracranial ICA calcifications in men, whereas hypertension and diabetes were found to be strong risk factors in women.^2^ This may indicate that either the protective effect of estrogen does not affect intracranial arteries as much as extracranial vessels, or that the effect of vascular risk factors on extracranial versus intracranial vessels differ between men and women. In a post-hoc analysis we did not find clear sex differences in vascular risk factors between patients with and without atherosclerosis but the numbers in the subgroups are very small (Supplementary Table 2). Although the overall intracranial atherosclerosis presence was similar for both sexes, women less frequently had intima calcifications compared with men. The prevalence of media calcifications on the other hand was higher in women, although this difference was no longer statistically significant after adjustment for confounders. The intimal layer consists of endothelial cells that proliferate in the process of atherosclerosis and grow into the arterial lumen forming plaques that narrow the lumen and can rupture. The medial layer consists of smooth muscle cells and elastic fibers which regulate blood flow and arterial pressure.^16^ The sex differences in intima calcifications could suggest a different pathophysiology of intracranial atherosclerosis in men and women. Possibly, a different vascular pathology than atherosclerosis, for example arterial stiffness, influences intracranial calcification.^17^

Our study has methodological limitations. First, intracranial ICA calcification volume could not be assessed in one-third of participants. The main reason for this was that due to technical reasons the thin slice NCCT could not be retrieved from two of the DUST centers. We considered these missing to be random. In addition, in a small number of participants NCCT scans could not be evaluated due to presence of artifacts. Furthermore, due to a multicenter design, the scans were performed in different hospitals possibly leading to inconsistency. Second, the sample size of our subgroup analysis of our young stroke patients was small. Therefore, these results should be interpreted with caution and differences between sexes cannot be ruled out because of limited power. Due to the small sample size of young stroke patients, we were not able to investigate pre- and post-menopause differences. Third, as our study was cross-sectional, we were not able to draw conclusions on sex differences in atherosclerosis development over time. In our cross-sectional setting we found no differences in presence of atherosclerosis between young men and women with stroke. Fourth, the DUST did not allow to include patients with severe renal disease who have a high burden of systemic atherosclerosis as renal dysfunction is a contraindication for CTA/CTP. Fifth, lack of ethnical diversity in our cohort causes a difficulty to generalize outside of western European populations. Lastly, in our study we focused on biological sex differences between men and women. Cardiovascular risk factors are probably also influenced by gender but in the DUST study no information on gender aspects was available.

Strong points are our large prospective cohort of Western European ischemic stroke patients who were all scanned within the first 9 h of stroke onset. In addition, we were able to use state of the art tools for volumetric assessment of the burden of intracranial atherosclerosis.

Future research should focus on pathophysiological mechanisms in sex differences behind development of intracranial and extracranial atherosclerosis preferably in longitudinal studies and in young populations. Sex differences might be most pronounced in this population due to the effects of sex hormones. This could further help understand differences in development of atherosclerosis between men and women, and may eventually lead to a more sex specific management and prevention of ischemic stroke.
